# An Analysis of Factors Affecting Genotyping Success from Museum Specimens Reveals an Increase of Genetic and Morphological Variation during a Historical Range Expansion of a European Spider

**DOI:** 10.1371/journal.pone.0136337

**Published:** 2015-08-26

**Authors:** Henrik Krehenwinkel, Stano Pekar

**Affiliations:** 1 Max Planck Institute for Evolutionary Biology, Department of Evolutionary Genetics, August Thienemann Strasse 2, 24306, Plön, Germany; 2 Department of Botany and Zoology, Faculty of Science, Masaryk University, Kotlářská 2, 611 37, Brno, Czech Republic; 3 University of California, Department of Environmental Science, Policy, and Management, 130 Mulford Hall, Berkeley, United States of America; Scientific Research Centre, Slovenian Academy of Sciences and Arts, SLOVENIA

## Abstract

Natural history collections house an enormous amount of plant and animal specimens, which constitute a promising source for molecular analyses. Storage conditions differ among taxa and can have a dramatic effect on the success of DNA work. Here, we analyze the feasibility of DNA extraction from ethanol preserved spiders (Araneae). We tested genotyping success using several hundred specimens of the wasp spider, *Argiope bruennichi*, deposited in two large German natural history collections. We tested the influence of different factors on the utility of specimens for genotyping. Our results show that not the specimen’s age, but the museum collection is a major predictor of genotyping success. These results indicate that long term storage conditions should be optimized in natural history museums to assure the utility of collections for DNA work. Using historical material, we also traced historical genetic and morphological variation in the course of a poleward range expansion of *A*. *bruennichi* by comparing contemporary and historical specimens from a native and an invasive population in Germany. We show that the invasion of *A*. *bruennichi* is tightly correlated with an historical increase of genetic and phenotypic variation in the invasive population.

## Introduction

Natural history museums provide a rich source for historical DNA studies. DNA from museum specimens can be used for taxonomic assignments [1–2], phylogenetic reconstructions [3–4], conservation biology [5], predator-prey interactions [6], or to trace historical genetic changes in populations [7–8]. However, when natural history collections were set up and until a few decades ago, the importance of deposited material for molecular studies was still unforeseen. Storage conditions aimed for a long term preservation of the specimen’s phenotype and not its DNA integrity [9]. For example, unsuitable preservatives or insecticides dramatically decrease the molecule’s lifetime [10]. Consequently, DNA from museum specimens is often degraded, broken apart into small pieces and present in much lower concentrations than in fresh samples [8]. Considering the degradation of historical DNA, mitochondrial sequences are usually the preferred genetic marker [11]. However, the use of nuclear DNA is inevitable, when fast evolving markers are needed to study recent genetic processes, e.g. population subdivisions in the context of contemporary range expansions [12].

Such range expansions and biological invasions have been frequently observed in recent decades and often attributed to global climate change or increasing global trade [13]. Population genetic analysis indicate a strong association of admixture of formerly isolated lineages and the success of such invasions [14–15]. Admixture could provide populations with novel adaptive variation, which might enable rapid phenotypic change and evolutionary responses and speed up expansion success. In native populations, on the other hand, novel variation might swamp existing adaptation and could be prevented by selection [16].

The European wasp spider *Argiope bruennichi* (Scopoli, 1772) has rapidly expanded its distributional range since about 1930, from warm Oceanic and Mediterranean regions into increasingly Continental climate zones [17]. Today it can be found as far north as Finland. Initial analysis indicated that this expansion is associated with genetic admixture and following adaptive divergence in expanding populations [12,18]. As a large and conspicuously colored spider, *Argiope bruennichi* is well represented in natural history collections and its expansion very well documented. The species is thus well suited to study historical genetic and phenotypic changes in the course of the expansion.

Here, we present a detailed study on the feasibility of PCR analysis of historical DNA from spiders. Natural history museums all over the world house huge arachnological collections, usually stored in ethanol, which is considered well suited for DNA preservation. Consequently, spiders are potentially promising targets for work on historical DNA. We present a comparative study of PCR and genotyping performance in ethanol preserved museum specimens of *Argiope bruennichi*. We obtained several hundred specimens from two large German natural history collections. In particular, we aimed to identify factors that are significantly associated with long term DNA integrity. We investigated, which variables affects the amplification success of mitochondrial- and nuclear DNA markers of different fragment lengths. By analyzing a large time series of several hundred historical and contemporary specimens from a native and an invasive population of *A*. *bruennichi*, we were also able to investigate genetic variation during the range expansion and associated phenotypic changes.

## Material and Methods

### Sample preparation, sequencing and genotyping

Samples were acquired from two large German natural history collections, the Senckenberg Museum in Frankfurt (182 specimens) and the Naturkundemuseum in Berlin (215 specimens) (see S1 Table in the Dryad repository for details of the specimens, http://dx.doi.org/10.5061/dryad.4th05). Permission to sample was granted by the curators. Adult specimens were examined under a Leica MZ95 stereomicroscope and their prosoma width was measured using an eyepiece ruler (Leica, Wetzlar, Germany). One leg of each specimen was removed with a heat sterilized forceps and then placed in pure ethanol. The spider leg was then allowed to dry at room temperature on a piece of tissue paper for a few minutes. Each leg was then cut into several pieces with a sterile razor blade. Then the tissue was transferred to lysis buffer and disrupted on a Tissuelyser by means of 5 mm long stainless steel beads (both Qiagen, Hilden, Germany) for 30s at 30 Hz. The Archivpure Cell & Tissue Kit (5PRIME, Hamburg, Germany) was used for the DNA extractions according to the manufacturer’s protocol. Modifications of the protocol include the increase of all reaction volumes by ½ and the substitution of glycogen solution (20 mg/mL, 5PRIME, Hamburg, Germany) as a DNA carrier during isopropanol precipitation. Moreover, we used only between 20–30 μl of hydratation solution (10 mM Tris, 1 mM EDTA, pH 7–8) to eventually dissolve the DNA pellet. A negative control extraction was added. Work with museum specimens was carried out in a different room than that on contemporary specimens to avoid DNA carryover.

Primer design was done using the primer 3 software [19]. Primers for the mitochondrial COI gene were designed based on an alignment of 1200 bp of contemporary sequences (see [12] and targeting two distinct fragment sizes of 130 and 350 bp. The respective PCR fragments both contained informative SNPs to distinguish the major mitochondrial haplogroups within the wasp spider. In addition to the mitochondrial DNA, we targeted four nuclear microsatellite fragments, two of approximately 150 and two of 250 bp, respectively (Primers MA53, MA55, MA56 & MA60 from [12]).

PCRs were run with 1 μl of the DNA extract on an Applied Biosystems Veriti Thermal Cycler (Applied Biosystems, Foster City, US), using the Qiagen Multiplex PCR kit (Qiagen, Hilden, Germany) with 40 cycles, according to the manufacturer’s protocol. A negative control reaction was added to each PCR. Positive PCRs served as a measure of DNA extraction success. Due to limited amount of DNA extract, we refrained from other quantification measures, e.g. gel electrophoresis or photometry. DNA sequencing and microsatellite genotyping followed the protocols described in [12]. DNA sequences were edited using the Codon Code Aligner software (Codon Code Corporation, Dedham, MA, USA). Microsatellites were called using Genemapper (Applied Biosystems, Foster City, USA). Genotyping success for a sample was defined as the generation of a readable sequence trace or a clearly defined microsatellite peak.

### Factors affecting genotyping success

We were interested in the effect of the following nine explanatory variables on the genotyping success: collection year, body size [mm], marker type (nuclear/mitochondrial), marker size (long/short), sex/stage (male/female/juvenile), distance of the museum collection to the sampling site [km], collection site, museum collection (Naturkundemuseum Berlin or Senckenberg Museum Frankfurt), and country of origin. As the design of the study was not orthogonal, i.e. some of these variables were strongly correlated, we excluded sex/stage, collection site, and country of origin, from the analysis. As several measurements (markers) were made on the same individual, we used Generalised Estimating Equations (GEE) with exchangeable correlation structure and binomial error structure. GEE estimates a population average model and accounts for repeated measures by specifying a working correlation structure [20]. The linear predictor included main effects and their two-way interactions. Multicolinearity among remaining variables was further resolved by centring the continuous explanatory variables. Beta coefficients (slope) were used to compare the effect of continuous explanatory variables (standardised by scaling prior to analysis) on the genotyping success. Effect size of categorical explanatory variables was estimated as odds ratio. The analysis was performed in R [21] using the geepack package [22].

### Genetic and phenotypic changes

We analysed a subset of published datasets of the mitochondrial COI gene, (~1200 bp) and morphological measurements (prosoma width) from an invasive and a native population (from [12]; see http://dx.doi.org/10.5061/dryad.r8n7c “Files: Sampling localities, Prosoma width, Alignment of complete historical COI sequences, Contemporary COI alignment”). While the latter study analysed the species’ range expansion on a wide geographic scale (across Europe), here we focused on two geographic regions, which are particularly well represented in our museum specimens: north-eastern Germany (primarily the states of Berlin and Brandenburg) and south-western Germany (primarily the states of Hessen, Rhineland Palatinate, and Baden-Württemberg). The latter correspond to the historical native range edge of the spider, while the former to the first records of invasion. Contemporary material from both regions is easily accessible, making these native and invasive populations particularly interesting to study temporal genetic changes.

We distinguished specimens collected into (1) before the range expansion (i.e. before 1930), (2) during the early phase of the expansion (> 1930 ≤ 1960), and (3) after the species had started to massively expand (> 1960). Due to little genotyping success in very old native populations, we analysed combined mitochondrial data for all specimens from before 1960 for South-western German populations. DNA sequences were aligned using MEGA 6 [23] under default alignment parameters. Using DNAsp [24], we calculated nucleotide diversity (and its standard deviation) for each time period and population. Morphological differentiation was also explored over the three time periods, focussing on the prosoma width as a proxy for body size.

## Results

### Factors, which affect genotyping success

**Fig 1:**
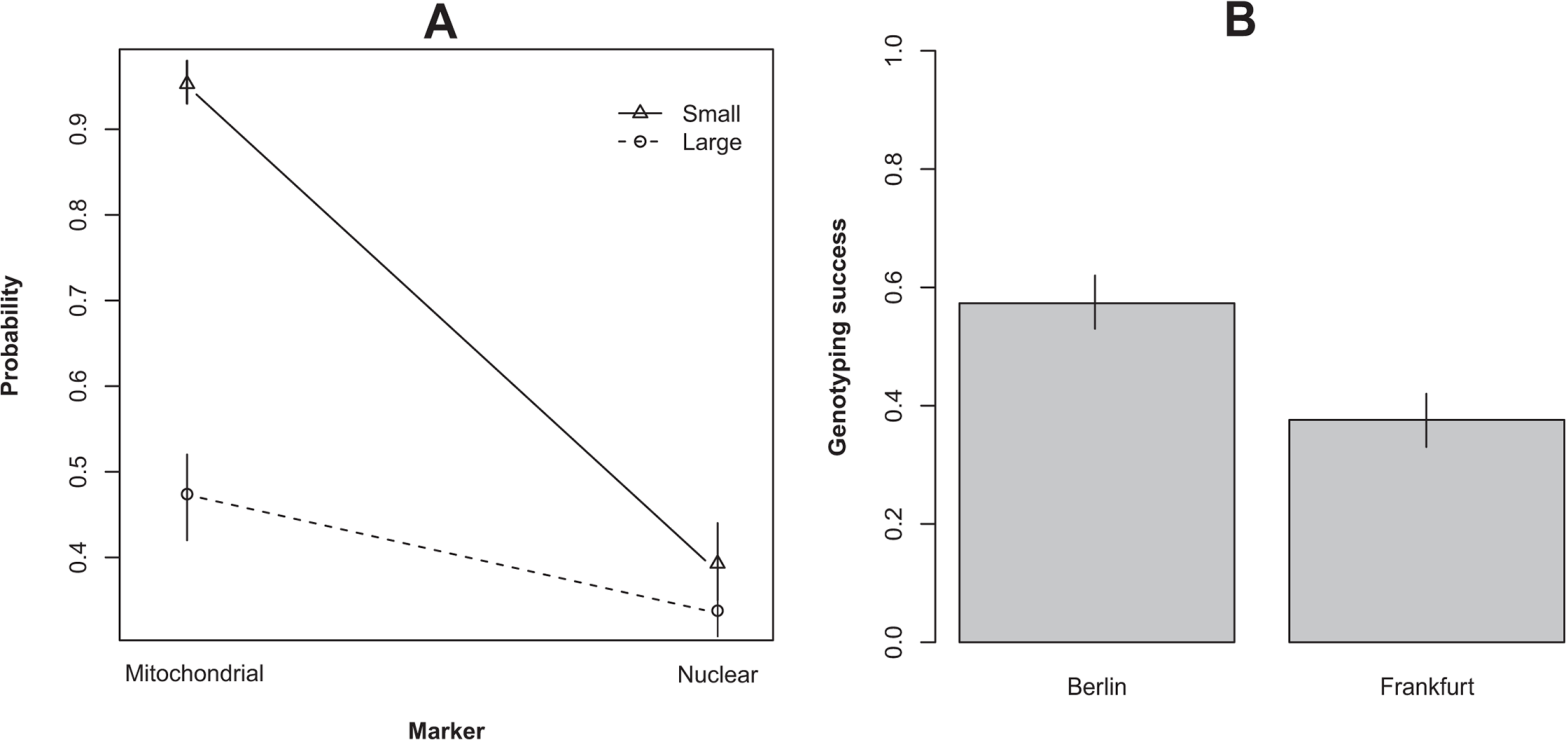
A. Comparison of probability of genotyping success for two types of markers and the size of the fragments. Points are means, vertical lines are 95% confidence intervals. B. Comparison of probability of genotyping success for the two museum collections. Bars are means, vertical lines are 95% confidence intervals.

We tested for the influence of six factors on genotyping success in 397 specimens. All study variables in interaction with one another had significant effect on the genotyping success (Table 1). The interaction between marker size and marker type was highly significant: both long and short nuclear markers were obtained with smaller chance than mitochondrial markers (Fig 1A). Long mitochondrial markers were obtained with 1.1 higher chance than long nuclear markers. But short mitochondrial markers were obtained with 5.1 times higher chance than short nuclear markers. Overall, short markers were obtained with 1.5 higher chance than long markers.

**Table 1:** Analysis of deviance table of the GEE model with main effects and two-way interactions.

Terms	Df	*X* ^2^	P
Year	1	15.4	< 0.0001
Bodysize	1	16.5	< 0.0001
Distance	1	0.4	0.53
Marker type	1	75.0	< 0.0001
Marker size	1	65.3	< 0.0001
Museum	1	41.6	< 0.0001
Year: Bodysize	1	0.4	0.52
Year: Distance	1	4.9	0.027
Year: Museum	1	0.6	0.44
Year: Marker type	1	0.0	1.0
Year: Marker size	1	1.5	0.22
Bodysize: Distance	1	5.7	0.017
Bodysize: Museum	1	0.5	0.47
Bodysize: Marker type	1	3.1	0.08
Bodysize: Marker size	1	3.9	0.047
Distance: Museum	1	1.1	0.29
Distance: Marker type	1	0.4	0.54
Distance: Marker size	1	0.1	0.74
Museum: Marker type	1	1.5	0.22
Museum: Marker size	1	2.2	0.13
Marker size: Marker type	1	26.4	< 0.0001

**Fig 2:**
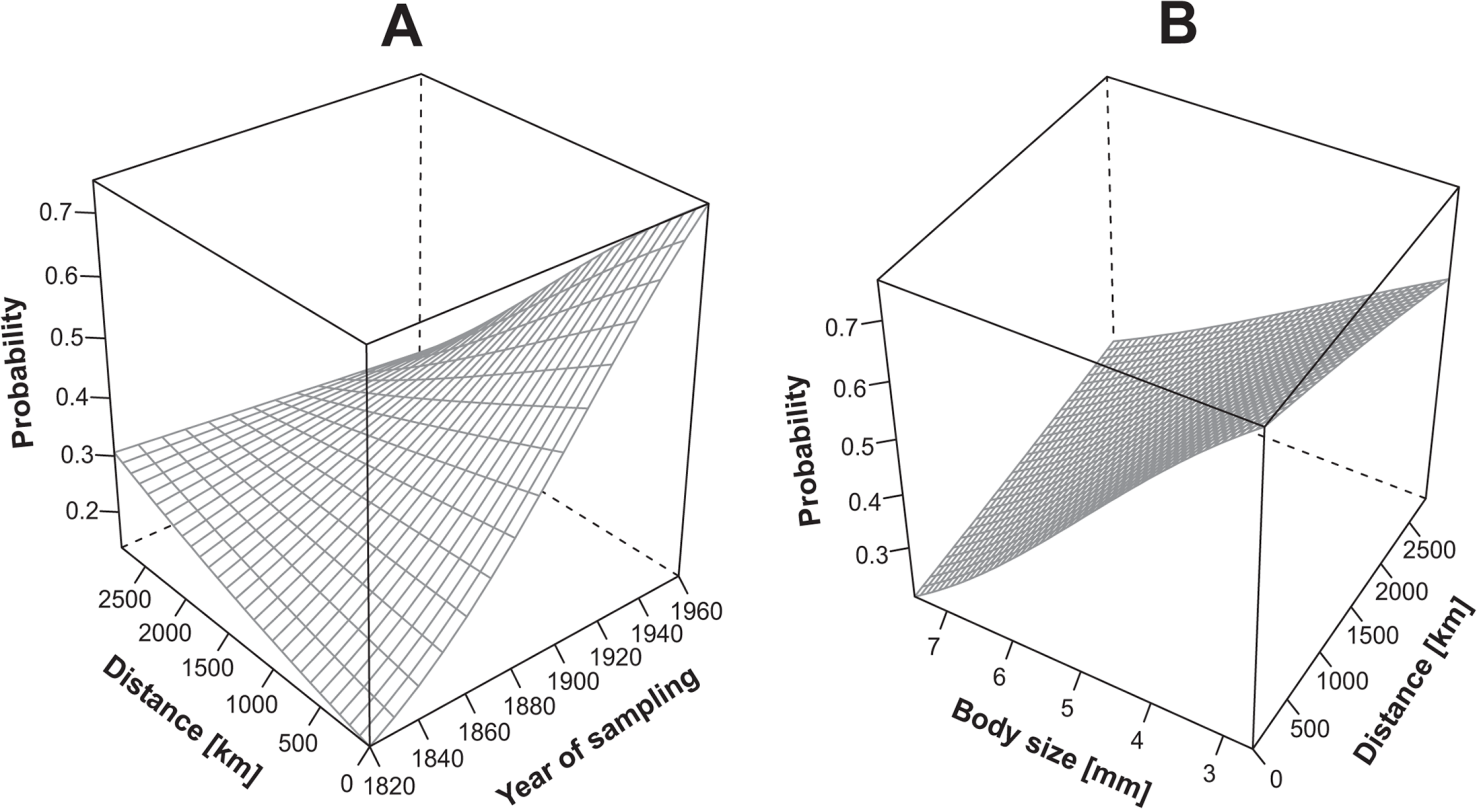
A. Relationship between the probability of genotyping success, distance of the sampling site to the museum collection and year of sampling. B. Relationship between the probability of genotyping success, distance of the sampling site to the museum collection and body size of spiders. Estimated logit models are shown.

The main effect of the museum collection was highly significant. Genotyping success, disregarding the marker type or size, was 4.5 times higher from material deposited in the Naturkundemuseum in Berlin than from material deposited in the Senckenberg Museum Frankfurt (Fig 1B). The interaction between distance to sampling site and year of sampling was also significant: the genotyping probability increased steeply with the year of sampling and increased very little with distance of the sampling site to the museum collection (Fig 2A). Finally, the interaction between distance of the museum collection to sampling site and body size was significant. The probability of genotyping decreased steeply with increasing body size (Fig 2B). Thus among continuous explanatory variables, body size had two-times stronger effect than year of sampling per unit change, followed by distance to sampling site.

### Genetic and phenotypic changes during a range expansion

**Fig 3:**
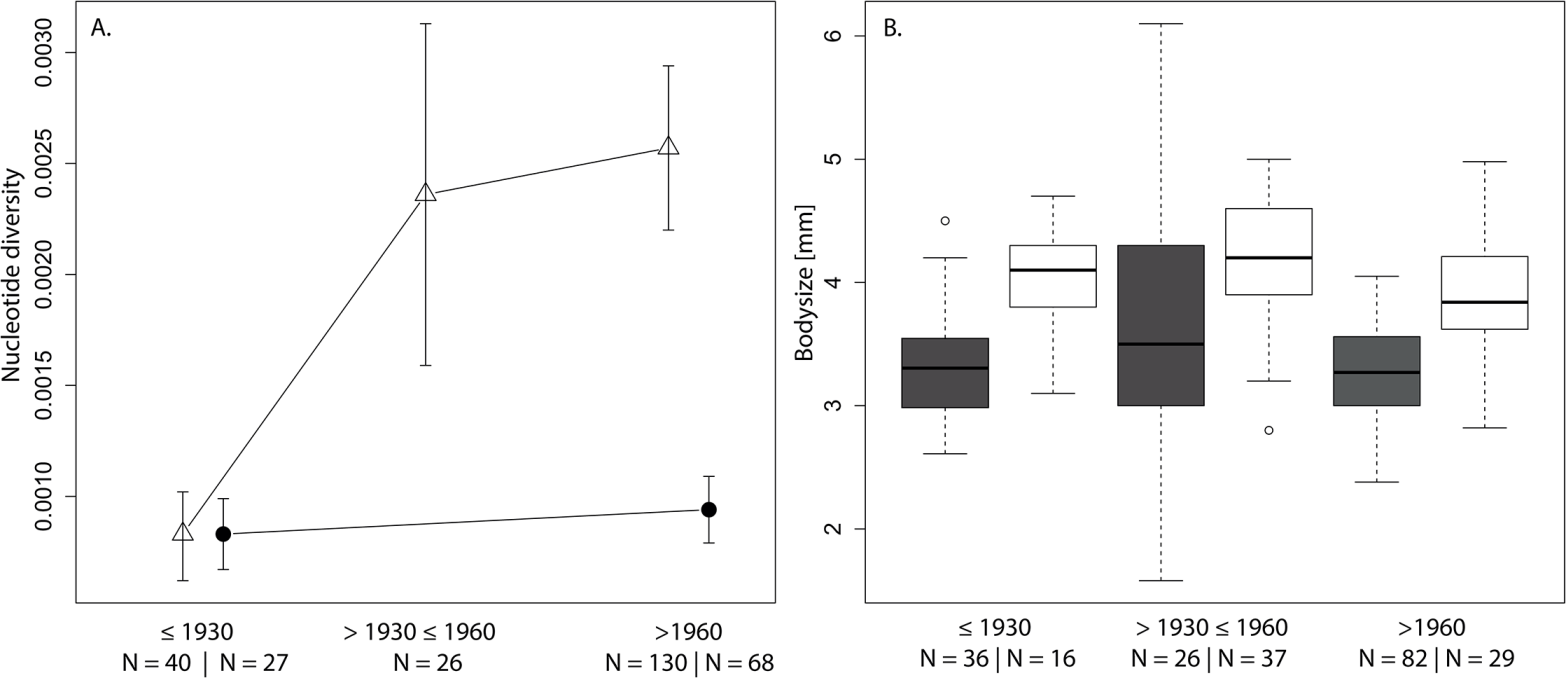
A. Comparison of nucleotide diversity (~1.2 kb of the mitochondrial COI gene) for invasive populations from north-eastern Germany (triangles) and native ones from south-western Germany (full circles) for three different time periods (time before the range expansion (≤ 1930), the early phase of the expansion (>1930≤1960), and the later massive expansion (>1960)). The whiskers are standard deviation. B. Comparison of the body size of specimens from the same populations (white = native & dark = invasive) over the same three time periods.

We analyzed contemporary and historical COI sequences of 291 specimens from the first invasive population of the species in north-eastern Germany and the historical native range edge in south-western Germany. Our analysis shows a clear association of increasing genetic variation and the ongoing invasion (Fig 3A). Before the onset of the range expansion, we find a comparably low diversity in both studied populations. With the onset of the expansion after 1930, we identify a sharp increase of genetic variation in invasive populations (2.8 fold increase). Diversity then plateaus in the invasive populations after 1960. Native populations, in contrast, retain largely similar low diversity values between historical and contemporary times.

In parallel to the increase of genetic variation after 1930, the body size variance of invasive populations increases markedly between 1930 and 1960 (Levene’s test, *p* < 0.001, Fig 3B). However, the variance of prosoma width has reduced again and is comparable to the time before 1930 (Levene’s test, *p* < 0.05). We did not find any significant change of body size variance over the studied time period in the native population (Levene’s test, *p* > 0.05). Although we found differences in morphological variation, we did not find significant changes of the mean body size in any of the studied populations (ANOVA, *p* > 0.05). However, invasive spiders are generally smaller than native ones (Welsh test, *p* < 0.001).

## Discussion

### Factors that affect genotyping success in historical spider specimens

Historical DNA can be routinely extracted and sequenced from preserved spiders, but spider DNA apparently suffers from pronounced degradation. Only short mitochondrial PCR fragments (only about 100 bp long) can be amplified in nearly 100% of samples. Molecular historical studies on spiders should thus rely on short PCR fragments. Indeed, such fragments can be sufficient for taxonomic or phylogeographic studies [1].

Due to higher copy number, mitochondrial fragments outperformed nuclear markers in our analysis. As expected, a specimen’s utility for molecular analysis does also depend on its age. However, the age was among less important predictors of genotyping success. We also did not observe a clear threshold after which a specimen is no longer useful for DNA analysis as has been found in insects [25]. Even an analysis of very old specimens, collected more than a hundred years ago, yielded positive results in many cases. Interestingly, smaller specimens were more likely to yield genotypes. A small spider might be simply more accessible to the preservative leading to quicker preservation and better DNA recovery. Interestingly, however, [26] discovered a positive association of bodysize and genotyping success in younger spider specimens. In younger specimens, the larger amount of tissue of larger spiders might simply outweigh the effects of DNA degradation. The strong effect of museum collection on the utility of a specimen for genotyping is quite surprising. Samples from the Naturkundemuseum Berlin performed much better in our analysis than those from the Senckenberg Museum Frankfurt. Spiders are usually stored in 70% ethanol and kept at room temperature in both these collections. However, the arachnological collections in Berlin and Frankfurt might have historically used different ethanol denaturants (e.g. camphor and methyl-ethyl-ketone, P. Jaeger, pers. communication), which might have influenced DNA integrity. Museum collections should consequently aim for a proper long term storage of samples. Using suitable preservatives, such as ethanol, a spider specimen will allow DNA extraction and genotyping more than a century after its collection. It is advisable to immediately place sampled spiders in ethanol, or to better remove a leg for storage in ethanol for optimal future DNA extraction. Due to their bilateral symmetry, the removal of a single leg will not alter the utility of a spider specimen for morphological analysis. Moreover, a separate storage of the leg will separate the tissue from the spider’s digestive fluids. Though pure ethanol generally is a well suited DNA and tissue preservative, arachnologist might consider other more favorable long term preservation methods. According to [27–28] propylene glycol outperforms ethanol in preserving DNA integrity in arachnids. Also, a storage at -20 or even -80°C is advised, for proper long term storage of DNA samples [28]. Propylene glycol is considerably cheaper than pure ethanol and might thus constitute a preferable alternative for spider preservation.

Considering the strong effect of museum collection on genotyping success, it is highly advisable to perform initial test extractions on samples, to estimate the utility of a whole collection for historical DNA analysis, before embarking on large scale genotyping projects.

### Genetic and phenotypic changes

The success of range expansions and biological invasions has often been associated with genetic admixture of formerly isolated lineages [14]. Such admixture might provide an invasive population with novel adaptive variation, which could facilitate a niche shift. This in turn might foster the colonization success [15]. Our results fit well with this scenario. The early expansion period is tightly associated with an increasing genetic variation in invasive populations. At the same time, we found a parallel increase of phenotypic variation (body size). The temporary increase of phenotypic variation might be a direct consequence of genetic admixture. Like the body size, other traits, e.g. thermal tolerance, could cause an increase in variance, from which a novel adaptive phenotype might be selected. Although *A*. *bruennichi* is a highly dispersive species, we did not find a comparable admixture and phenotypic change in native populations. This is surprising, as the analyzed south-western and north-eastern German populations are located next to each other. A selection against introgressing variation might be causative for the observed difference. While novel variation might be advantageous in continental Northern European climate, it could swamp adaptations in native populations [16]. However, due to demographic effects, the expanding populations might have simply been more prone to admixture [29].

## Supporting Information

S1 Table Summary of analyzed museum specimens and associated factors. Genotyping success is indicated by 1 for success and 0 for failure.
(XLSX)
